# REFRAMING AGING: INTRA- AND INTERGENERATIONAL DIGITAL CONVERSATIONAL AGENTS TO SUPPORT OLDER ADULTS

**DOI:** 10.1093/geroni/igac059.281

**Published:** 2022-12-20

**Authors:** Molly Han, Cameron Piercy

**Affiliations:** University of Kansas, Lawrence, Kansas, United States; University of Kansas, Lawrence, Kansas, United States

## Abstract

Digital conversational agents (DCAs) have become extraordinarily ubiquitous. Researchers envision the prospects of using DCAs to monitor health among older adults. However, older adults show hesitation to engage with DCAs. It is possible older adults prefer receiving human assistance rather than getting help from a machine. Another potential explanation is that communicative cues of DCAs such as voice need to be further optimized to invoke behavioral engagement. To understand how DCAs can better support older adults, we develop an experiment with three scenarios in which an agent (a human, an embodied DCA, a mixed presence of human and DCA) shares active aging information. We manipulate the agent’s voice in terms of age (older voice, younger voice). We investigate how the interplay of agent categories and intragenerational/intergenerational voice cues affect older adult participants’ evaluation of information and intention to adopt DCAs. Our study will contribute to DCAs design for older clients.

